# ESM-1 siRNA Knockdown Decreased Migration and Expression of CXCL3 in Prostate Cancer Cells

**Published:** 2017-03

**Authors:** Juan Rebollo, Jan Geliebter, Niradiz Reyes

**Affiliations:** 1 Research group of Genetics and Molecular Biology. School of Medicine. University of Cartagena. Colombia;; 2 Graduate School of Basic Medical Sciences. New York Medical College. Valhalla, NY, United States;; 3 Research group of Genetics and Molecular Biology. School of Medicine. University of Cartagena. Colombia

**Keywords:** endocan, chemokines, ESM-1 knockdown, migration, prostate cancer, proteoglycans

## Abstract

Endothelial cell-specific molecule-1 (ESM-1), also known as endocan, is a soluble proteoglycan expressed by the vascular endothelium, which also circulates in the bloodstream. Inflammatory cytokines and proangiogenic growth factors increase its expression, and increased serum levels have been reported in several cancer types and immunocompetent patients with sepsis. The aim of this study was to analyze the expression profile of CXC-chemokines and the effects of ESM-1 gene knockdown in proliferation, migration and CXC-chemokine expression in highly metastatic human prostate PC-3 cells. Expression profiles of CXC-chemokines were analyzed in metastatic PC-3 and non-tumorigenic PWR-1E cells. siRNA-mediated knockdown of ESM-1 was performed into PC-3 cells, which were subsequently tested for cell migration and proliferation. Effect of siRNA transfection on CXC-chemokine expression was further quantified at the transcript and protein level. RT-qPCR analysis and sandwich ELISA assay revealed higher levels of ESM-1 and several CXC-chemokines in metastatic PC-3 cells compared to non-tumorigenic PWR-1E. Transfection of PC-3 cells with ESM-1-siRNA decreased cell migration with no effect on proliferation, and it was accompanied by decrease in the transcript and protein levels of the angiogenic chemokine CXCL3. We report here for the first time the ESM-1 targeting in PC-3 cells, which resulted in decreased migration, which may be related, at least in part, to decreased expression of the angiogenic CXCL3 chemokine, whose expression was found to be reduced in ESM-1-siRNA transfected cells. Additional studies are required to ascertain the biological role of ESM-1 in prostate cancer cells and the link with the expression of CXCL3.

## INTRODUCTION

Endothelial cell-specific molecule-1 (ESM-1), also known as endocan, is a secreted proteoglycan whose transcripts were initially detected from a variety of cultured human endothelial cells of different origins, including coronary, pulmonary artery, dermal, and capillary endothelial cells (1, 2) . More recent studies have reported its expression by epithelial tumor cells and cancer cell lines, such as prostate cancer cells (3, 4) , melanoma cells (5, 6) , and glioblastoma cell lines(7). It has been found also in the blood circulation of healthy subjects (8) , and increased levels have been reported in patients with different pathologies, including inflammatory bowel disease (9), hepatocellular carcinoma (10), acute myeloid leukemia (11) , among others.

ESM-1 is regarded as a marker of angiogenesis, an important factor in the progression of cancer (12). Its transcriptional expression has been shown to be regulated by angiogenic growth factors, such as vascular endothelial growth factor (VEGF), and by inflammatory cytokines, such as tumor necrosis factor alpha (TNF-α) (13).

Angiogenesis is a process shown to be regulated by different factors, produced by both tumor cells and host responding cells (14). Among the regulatory factors, increasing evidence supports the important role of CXC chemokines in the process of angiogenesis (15, 16) . The majority of CXC chemokines contain at the NH_2_ terminus a three-amino-acid motif (Glu-Leu-Arg: the ELR motif), which precedes the first cysteine amino acid of the primary structure of these cytokines (17). The CXC chemokine family members that contain the ELR motif (ELR+) are potent promoters of angiogenesis (18) , while members that lack the ELR motif (ELR−) are potent inhibitors of angiogenesis, or angiostatic (18, 19).

We have previously reported the over-expression of ESM-1 in prostate cancer cell lines from rat (3) and human origin (4). The aim of this work was to analyze the expression profile of CXC-chemokines and the effects of ESM-1 gene knockdown in proliferation, migration and CXC-chemokine expression in highly metastatic human prostate PC-3 cells.

## MATERIALS AND METHODS

### Cultured cell lines

Human bone metastasis-derived prostate cancer PC-3 cells (ATCC^®^ CRL-1435) and non-tumorigenic human prostatic epithelial PWR-1E cells (CRL-11611) were obtained from the ATCC (Manassas, VA). PC-3 cells were routinely maintained in phenol red-positive F-12K modified medium (ATCC) containing 10% FBS and 1% penicillin-streptomycin. PWR-1E cells were maintained in keratinocyte serum-free medium (Life Technologies) supplemented with 50 μg/mL bovine pituitary extract, 5% L-glutamine, and 5 ng/mL epidermal growth factor. Cells were grown as monolayers in T-25 tissue culture flasks, in a humidified atmosphere containing 5% CO2 at 37°C and passaged once/twice a week. For all the experiments, cells were harvested at low passage numbers: PC-3 cells between passages 28 and 31, and PWR-1E between passages 18 and 22.

### siRNA-mediated knockdown of ESM-1 gene expression

Small interfering RNA targeting the ESM-1 gene (siESM-1) and scramble siRNA sequence (siControl), were purchased from Qiagen (FlexiTube GeneSolution GS11082 for ESM-1). PC-3 cells were seeded into 12-well cell culture plates (Corning^®^) and incubated for 24 h in Opti-MEM^®^ reduced serum medium (GIBCO) without FBS. Then, cells were subjected to transfection with either siESM-1 or siControl using the RNAi Human/Mouse Starter Kit (Qiagen) following the manufacturer's instructions. Briefly, 300,000 cells were mixed with siRNAs solution complexed with the transfection reagent, and the mixture was incubated at normal culture conditions for 48 hours. Non-transfected (NT) cells treated in the same way were used as control.

### Cell viability and proliferation assays

To measure the cell survival and proliferation rate of PC-3 cells, after treatment with siESM-1 or siControl, cell viability and proliferation were analyzed 48 h post-transfection using two methods: a direct cell counting using trypan blue dye exclusion and a colorimetric MTS-based cell viability kit (CellTiter 96 Aqueous One Solution Cell Proliferation Assay, Promega^®^). For trypan blue dye exclusion method, PC-3 cells transfected with siRNAs were mixed with 10 ul trypan blue (0.4% in PBS) and the number of viable, unstained cells, was determined using a Neubauer chamber. For the MTS-based assay, PC-3 cells transfected with siRNAs were mixed with 20 μL CellTiter solution (Promega) and incubated for an additional 4 h at 37°C. Then, absorbance was measured at 490 nm in a Multiskan FC microplate reader (Thermo Scientific). MTS [3-(4,5-dimethylthiazol-2-yl)-5-(3-carboxymethoxyphenyl)-2-(4-sulfophenyl)-2H-tetrazolium, inner salt] is a tetrazolium salt that is reduced to water-soluble formazan mainly by mitochondrial dehydrogenase enzymes found in metabolically active cells. The quantity of formazan product as measured by the amount of 490nm absorbance is directly proportional to the number of living cells in culture (20).

### Scratch assay for analysis of cell migration *in vitro*

To evaluate the effect of ESM-1 gene expression knockdown in cell migration, the scratch assay was performed (21). PC-3 cells were seeded onto 6-well plates at density of 3 × 10^6^ cells/well, and incubated at 5% CO_2_ and 37°C for 24 h. Then, cells were transfected with ESM-1-siRNA or control-siRNA, and incubated for additional 24h. Media was subsequently replaced by serum-free Opti-MEM and cells were incubated until they reached 90-100% confluence. At this point, a vertical scratch was performed on PC-3 cells monolayer using a sterile 1,000 μl micropipette tip. Cells were carefully washed with Opti-MEM to remove scratched cells, and further incubated at 37°C in fresh Opti-MEM. Wound closure was monitored every 12 h post scratch with a trinocular inverted microscope (Motic AE31). Images were analyzed with the TScratch software (22). Experiments were performed in triplicates. Untransfected and control-siRNA transfected cells were used as controls.

### Total RNA extraction and quality control

Untransfected PWR-1E cells were used as the reference cell line for comparison of gene expression between transfected and untransfected conditions. To analyze the effects of siRNA transfection in gene expression, control-siRNA transfected PC-3 cells were used as control. Total RNA was isolated from PC-3 cells on the following conditions: untransfected, ESM-1-siRNA transfected, and control-siRNA transfected. RNA isolation was performed with TRI-Reagent (Ambion), following the manufacturer’s instructions. The concentration and purity of the extracted total RNA was assessed spectrophotometrically by measuring OD260 and OD260/280 ratio respectively in RNase-free H_2_O using Nanodrop 2000c (Thermo Scientific). Three replicates of each RNA sample were measured and the measured concentrations were averaged. For all samples OD260/280 ratio was ~2.0. Total RNA samples were stored at −80°C until used.

### cDNA synthesis and reverse transcription quantitative real-time PCR (RT-qPCR)

RNA samples from cell lines were processed for reverse transcription using the QuantiTect Reverse Transcriptase kit (Qiagen). First, to eliminate any contaminating genomic DNA, 1μg of total RNA was incubated with genomic DNA Wipeout Buffer (Qiagen) for 2min at 42°C. The reverse-transcription master mix, containing QuantiTect RT enzyme, QuantiTect RT Buffer and RT Primer Mix (oligo-dT and random primers), was prepared and added to the template RNA. Samples were incubated at 42°C for 15 min, followed by inactivation at 95°C for 3 min. A 20 μL final volume of cDNA was stored at −20°C until used in qPCR.

Transcript expression levels for ESM-1, ELR+ CXC chemokines (CXCL1, CXCL2, CXCL3, CXCL5, CXCL6, CXCL7, and CXCL8), ELR− CXC chemokines (CXCL4, CXCL9, CXCL10, CXCL11, CXCL12, and CXCL14), and reference genes HPRT-1 and *β*-actin were analyzed in the cell lines by qPCR using QuantiTect SYBR Green PCR Master Mix (Qiagen). Primers specific for ESM-1 and the reference genes HPRT-1 and *β*-actin were designed with Primer-BLAST (23) using sequences from GenBank database. All primers were designed to span at least one exon–intron boundary to avoid detection of residual genomic DNA. For gene expression analysis of CXC-chemokines, Chemokines qSTAR qPCR primer panels (HPP6004A) were purchased from OriGene Technologies (Rockville, MD). qPCR reactions for each sample were carried out in triplicates, using QuantiTect^®^ SYBR^®^ Green PCR Master Mix (Qiagen) in a StepOne thermocycler (Applied Biosystems), with an initial denaturing step at 95ºC for 15 min, followed by 40 cycles of amplification of 95 °C for 15 s, 55 °C for 45 s and 60°C for 1 s. Negative controls (non-template control and negative reverse transcriptase control) were also included. Relative changes in gene expression for each target gene in the cell lines were calculated with the Sequence Detection System 2.1 software (Applied Biosystems), using the comparative C_T_ method (2^–ΔΔCT^). Expression levels for each target gene were normalized to the expression levels of the reference genes HPRT1 and β-Actin. Melting curves for all samples were acquired for quality control purposes.

### Enzyme-linked immunosorbent assay for CXC Chemokine measure

Supernatants were collected from cells cultured in serum free medium for 48 hours. Cell supernatants were cleared by centrifugation at 13,000 rpm for 5 min. Protein level for each chemokine was measured by quantitative enzyme-linked immunosorbent assay (ELISA). CXCL1/GROα, CXCL8/IL8, CXCL9/MIG, CXCL10/ IP-10, and CXCL11/I-TAC protein levels were determined using the Human Common Chemokines Multi-Analyte ELISArray Kit (SABiosciences, Qiagen, Valencia, CA, USA), while CXCL3 protein level was determined with a single ELISA assay kit (Cat #: MBS910472, Mybiosource, San Diego, CA, USA). Briefly, 50 µL of medium was incubated in a 96-well plate for 1 hour at RT, followed by washing steps, incubation with detection antibodies for 1 hour, washing, incubation with avidin-horseradish peroxidase for 30 minutes, washing, development for 15 to 30 minutes, and termination of the development with stop solution. Chemokine protein levels were measured on a MultiSkan microplate reader (Thermo) with an absorbance at 450 nm. Sampling was performed in triplicates.

### Enzyme-linked immunosorbent assay for ESM-1 measure

Supernatants were collected from cells cultured in serum free medium for 48 hours. Cell supernatants were cleared by centrifugation at 13,000 rpm for 5 min. ESM-1 protein levels were measured using a commercial enzyme-linked immunosorbent assay (Aviscera Biosciencies, Santa Clara, CA, USA) according to the manufacturer’s instructions. A microwell plate was coated with 100 μl of capture antibody (1 µg/ml) against ESM-1, incubated overnight at 4°C, and then coated again with 1% BSA. Then, 100 μl of serially diluted ESM-1 standard solution included in the ELISA kit, and samples diluted fivefold in dilution buffer (2% BSA in PBST) were applied to wells in triplicate and incubated with 0.25 μg/ml detection antibody for 1 h, at room temperature. After washing the wells with PBST solution, 100 μl of diluted HRP-conjugated streptavidin was applied to each well for 30 min. Subsequently, tetramethylbenzidine (TMB) solution was added to the wells, and then the reaction was stopped with 1 N H_2_SO_4_ solution. The absorbance was then measured at 450 nm on a MultiSkan microplate reader (Thermo).

### Statistical analysis

For relative mRNA expression quantification, the comparative CT method (2^–ΔΔCT^) was used, and expression levels for each sample were normalized to the expression level of the reference genes HPRT1 and β-Actin. qPCR experiments were conducted in triplicate. A *p*<0.05 was considered statistically significant. For single ESM-1 and CXCL3 ELISA assays and for multi-analyte ELISA array, data were reported as mean ± SEM. Statistical difference in gene expression for ESM-1 and for each chemokine, between PC-3 prostatic cancer cell line and the control cell line, and between PC-3 cells transfected with ESM-1-siRNA and PC-3 cells transfected with control-siRNA, was calculated with the T-test using GraphPad Prism 5 software (GraphPad Software Inc, San Diego, CA); *p*<0.05 was considered statistically significant.

## RESULTS

### ESM-1 and CXC chemokine expression in PC-3 prostate cancer cells

**Figure 1:**
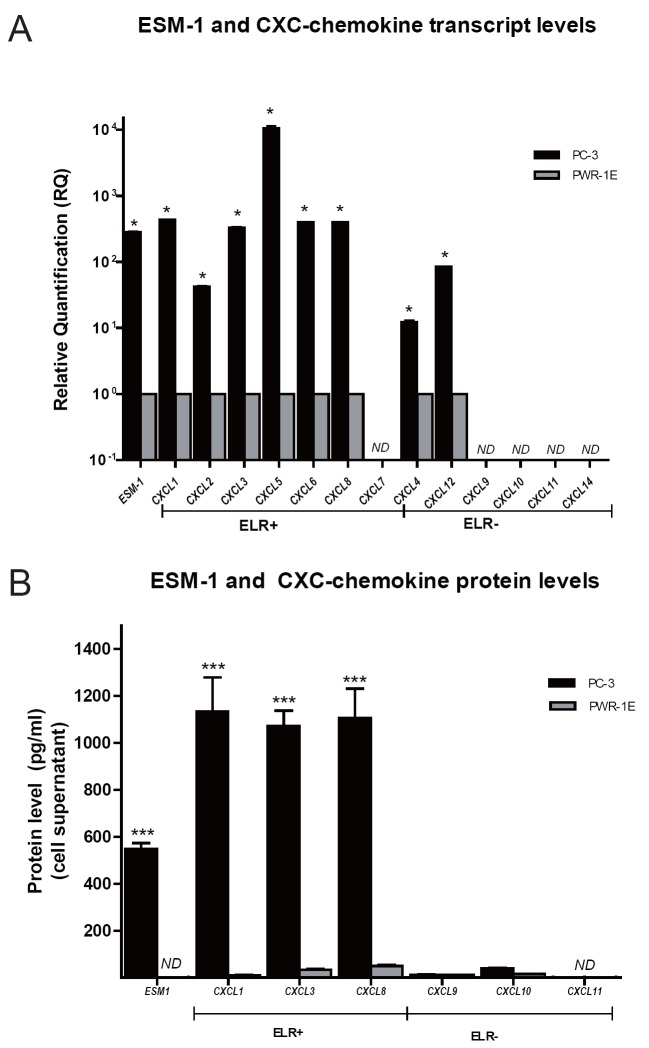
ESM-1 and CXC-chemokine expression in PC-3 cells relative to non-tumorigenic PWR-1E cells. A, Transcript expression levels for ESM-1, ELR+ CXC chemokines (CXCL1, CXCL2, CXCL3, CXCL5, CXCL6, CXCL7, CXCL8), and ELR− CXC chemokines (CXCL4, CXCL9, CXCL10, CXCL11, CXCL12, CXCL14), were analyzed in the cell lines by RT-qPCR. Relative changes in gene expression for each target gene were calculated using the comparative CT method (2^–ΔΔCT^) normalizing to the expression levels of reference genes (HPRT-1 and *β*-actin). Greater than twofold expression changes were regarded as significant and are marked by asterisks; B, Protein levels were measured using quantitative commercial enzyme-linked immunosorbent assay/ELISA for ESM-1 and CXC chemokines, according to the manufacturer's instructions. Chemokines evaluated were CXCL1/GROα, CXCL3, CXCL8/IL8, CXCL9/MIG, CXCL10/IP-10, and CXCL11/I-TAC. ESM-1 and CXC chemokine levels in cell supernatants are shown as mean ± SEM.

Transcript and protein expression data for ESM-1 and CXC chemokines are presented in Figure 1. Overall, qPCR results showed that, compared to non-tumorigenic PWR-1E cells, metastatic PC-3 cells overexpressed transcripts encoding ESM-1 and transcripts encoding the ELR+ CXC chemokines CXCL1, CXCL2, CXCL3, CXCL5, CXCL6, and CXCL8. Conversely, transcripts encoding the ELR− CXC chemokines CXCL9, CXCL10, CXCL11, and CXCL14 were not detected in either PC-3 or PWR-1E cells (Figure 1A). At the protein level, ESM-1 ELISA assay confirmed the high expression level of ESM-1 in the supernatant of cultured PC-3 cells. ESM-1 was not detected in the supernatant of the control non-tumorigenic PWR-1E cells. Multiplex and single ELISA assays confirmed the differential CXC chemokine profiles between PC-3 cells and the control non-tumorigenic PWR-1E cells. Culture medium from PC-3 cells had high levels of ELR+ CXC chemokines CXCL1, CXCL3, and CXCL8 but low levels of ELR- CXC chemokines CXCL9 and CXCL10, and non- detected levels of CXCL11. In contrast, the non-tumorigenic PWR-1E cells had low levels of both ELR+ and ELR- CXC chemokines (Figure 1B). These results are consistent with the highly metastatic phenotype of PC-3 cells and the non-tumorigenic phenotype of PWR-1E cells.

### ESM-1 and CXC chemokine expression of siRNA transfected PC-3 cells

**Figure 2:**
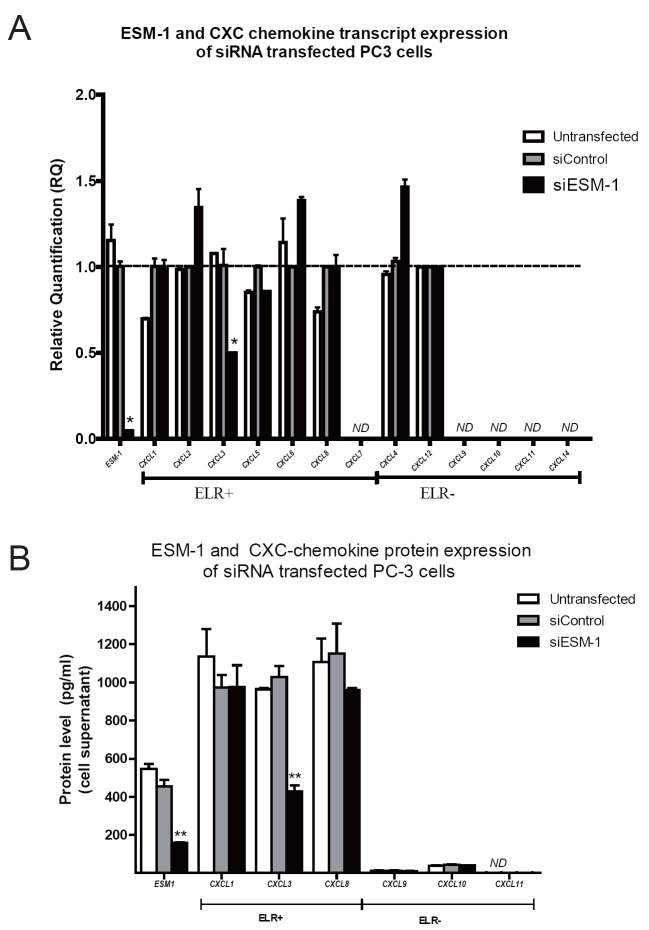
ESM-1 and CXC chemokine expression of siRNA transfected PC-3 cells. A, At 48 h post transfection, transcript level of ESM-1 in cells transfected with ESM-1 siRNA was significantly reduced compared with cells transfected with control siRNA. Knockdown of ESM-1 in PC-3 cells did not affect the transcript expression of most CXC chemokines, except for CXCL3. ESM-1 silencing was associated to decreased transcript levels of CXCL3 chemokine, compared to cells transfected with control siRNA or untransfected cells; B, Knockdown of ESM-1 in PC-3 cells significantly reduced the ESM-1 protein level and it was associated to decreased level of CXCL3. ESM-1 knockdown did not affect the protein expression of the remaining CXC chemokines evaluated. Chemokines evaluated were CXCL1/GROα, CXCL3, CXCL8/IL8, CXCL9/MIG, CXCL10/ IP-10, and CXCL11/I-TAC. ESM-1 and CXC chemokine protein levels in cell supernatants are shown as mean ± SEM. *siControl: PC-3 cells transfected with control siRNA.siESM-1: PC-3 cells transfected with ESM-1 siRNA for 48 h.*

ESM-1 and CXC-chemokine genes, which are implicated in invasion and migration, were analyzed at the transcript and protein levels by qPCR and ELISA, respectively, in PC-3 cells transfected with either ESM-1-siRNA or control-siRNA (Figure 2). As shown in Figure 2A, at 48 h post transfection, the transcript level of ESM-1 in cells transfected with ESM-1 siRNA were significantly reduced compared with cells transfected with control siRNA. Knockdown of ESM-1 in PC-3 cells did not affect the transcript expression of most CXC chemokines, except for CXCL3. PC-3 cells transfected with ESM-1 siRNA had significantly lower transcript levels of CXCL3 compared to PC-3 cells transfected with control-siRNA. Decreased expression of CXCL3 was specific for ESM-1 siRNA transfected PC-3 cells since there was no significant difference in CXCL3 transcript expression in untransfected PC-3 cells compared to PC-3 cells transfected with control-siRNA. CXCL7, CXCL9, CXCL10, CXCL11 and CXCL14 were non detected (ND) in PC-3 cells. There was no significant difference in the transcript expression of the remaining CXC chemokines associated to the knockdown of ESM-1 in PC-3 cells. At the protein level, expression of ESM-1 and CXCL3 in cells transfected with ESM-1 siRNA was significantly reduced compared with cells transfected with control siRNA. Knockdown of ESM-1 in PC-3 cells did not affect the protein expression of the remaining CXC chemokines evaluated: CXCL1/GRO CXCL8/IL8, CXCL9/MIG, CXCL10/ IP-10, and CXCL11/I-TAC (Figure 2B).

### Effect of ESM-1 siRNA knockdown in viability and proliferation of PC-3 cells

**Figure 3:**
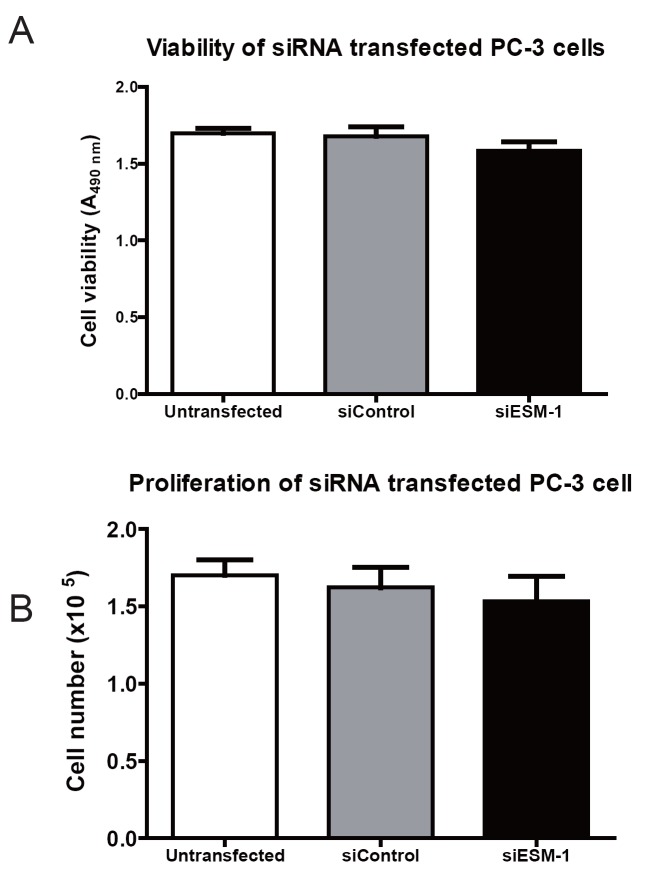
Cell viability and proliferation of siRNA transfected PC-3 cells. Cell survival (A) and proliferation rate (B) of PC-3 cells transfected with siESM-1 or siControl, were analyzed 48 h post-transfection using a MTS-based colorimetric test and direct cell counting trypan blue dye exclusion, respectively. Knockdown of ESM-1 did not affect cell viability or proliferation of PC-3 cells. siControl: PC-3 cells transfected with control siRNA. siESM-1: PC-3 cells transfected with ESM-1 siRNA.

Cell viability was evaluated using the direct cell counting trypan blue exclusion (A) and the MTS-based cell titer viability assay (B). Neither the viability (Figure 3A), nor the proliferation (Figure 3B), of PC-3 cells transfected with ESM-1 siRNA were significantly different to that of PC-3 cells transfected with control siRNA. Forty-eight hours after transfection, cell viability and cell proliferation was 94.4% in ESM-1-siRNA transfected PC-3 cells compared to PC-3 cells transfected with control-siRNA. Thus, ESM-1 siRNA knockdown did not significantly affect cell proliferation of PC-3 cells.

### Effect of ESM-1 siRNA knockdown in migration of PC-3 cells

**Figure 4:**
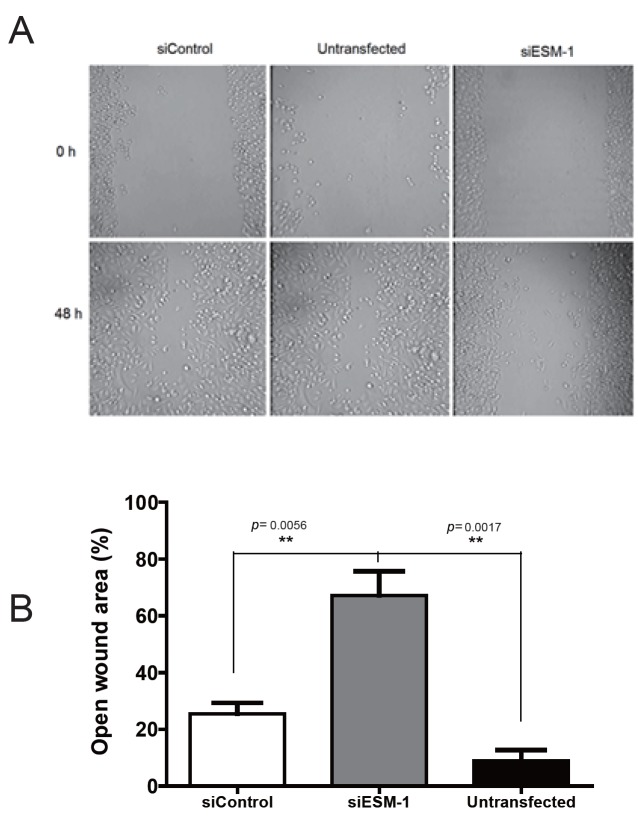
Cell migration of siRNA transfected PC-3 cells. Cell migration of PC-3 cells was assayed with the scratch “wound healing” assay. Knockdown of ESM-1 in PC-3 cells inhibited migration *in vitro*. siControl: PC-3 cells transfected with control siRNA. siESM-1: PC-3 cells transfected with ESM-1 siRNA.

A wound healing assay revealed that 48 h after scratch treatment, the open wound area of ESM-1 siRNA-transfected cells was significantly larger compared to that of control-siRNA-transfected or untransfected cells (Figure 4). Thus, knockdown of ESM-1 in PC-3 cells inhibited cell migration *in vitro*.

## DISCUSSION

Prostate cancer is the most frequently diagnosed non-skin cancer in the United States (24) and the incidence of metastatic prostate cancer has increased in the last decade in this country (25). Increasing efforts have been addressed to understand the biology of this disease. Endocan is a soluble proteoglycan that is overexpressed by metastatic cell lines from different origins, including prostate (3, 4), glioblastoma (7) , colon cancer (26) , among others. It has been also found circulating in the bloodstream of healthy individuals, patients with inflammatory diseases and cancer patients (9, 11, 27) . Increasing evidence shows the involvement of this proteoglycan in the control of fundamental cellular processes such as adhesion, migration and angiogenesis (28) . Among these, angiogenesis is central for cancer progression, and it is regulated by both activator and inhibitor molecules, including endocan and CXC chemokines, which are increasingly shown to play a key role in angiogenesis of cancer (28, 29). So far, the role of endocan in prostate cancer remains unknown.

In the present study we investigated whether siRNA-mediated silencing of endocan expression affected proliferation, migration and/or CXC chemokine expression of PC-3 cells. We found that siRNA-mediated silencing of endocan expression in the highly metastatic prostate cancer cell line PC-3 results in decreased migration with no effect on cell proliferation. Consistent with previous results in other cell types (30, 31) , endocan silencing in PC-3 cells resulted in decreased cell migration in *in vitro* assays. Since endocan is regarded as a marker of angiogenesis, a process that has been shown to be regulated by several factors, including the CXC chemokine family (15) , we further examined whether endocan knockdown in these cells affected the mRNA expression of CXC chemokines. We found that endocan gene-silencing in PC-3 cells was accompanied by decreased expression of CXCL3, a member of the angiogenic ELR+ CXC chemokine group.

CXCL3 and its receptor CXCR2 have been recently found overexpressed in prostate cancer cells, prostate epithelial cells and prostate cancer tissues, which may implicate a role for this chemokine in prostate cancer progression and metastasis (32). CXCL3 is a member of the CXC chemokine family and it is sub-classified as a Glu-Leu-Arg (ELR+) CXC chemokine (33). CXCL3 has been found previously over-expressed in the aggressive PC-3 cell line and its tissue expression correlates with prostate cancer metastasis (32, 34) . Our results show that ESM-1 targeting in PC-3 cells resulted in decreased migration, which may be related, at least in part, to decreased expression of the angiogenic CXCL3 chemokine whose expression was found to be reduced in endocan siRNA transfected cells. CXCL3 has been shown to act as a chemoattractant for neutrophils to areas of brain injury (35) and for cerebellar progenitor cells (36) , while it is not clear for prostate cancer cells if this chemokine is chemoattractant or not (32). The results shown here deserve further investigation. Additional studies are required to determine the mechanisms underlying the decreased expression of CXCL3 in endocan siRNA silenced PC-3 cells, and more research is needed to ascertain the biological role of ESM-1 in prostate cancer. It will be important to determine the effects of ESM-1 knockdown in other recognized angiogenic markers and the effects of ectopically expressing ESM-1 in cells with ESM-1 knockdown.
